# Financial Burden and Impoverishment Due to Cardiovascular Medications in Low and Middle Income Countries: An Illustration from India

**DOI:** 10.1371/journal.pone.0155293

**Published:** 2016-05-09

**Authors:** Kiran Raj Pandey, David O. Meltzer

**Affiliations:** Section of Hospital Medicine, Department of Medicine, University of Chicago, Chicago, Illinois, United States of America; McGill University Health Center / Royal Victoria, CANADA

## Abstract

**Background:**

Health expenditures are a major financial burden for many persons in low and middle-income countries, where individuals often lack health insurance. We estimate the effect of purchasing cardiovascular medicines on poverty in low and middle-income populations using rural and urban India as an example.

**Methods:**

We created step-up treatment regimens for prevention of ischemic heart disease for the most common cardiovascular medications in India based on their cost and relative risk reduction. Cost was measured by Government of India mandated ceiling prices in rupees (Rs. 1 = $0·016) for essential medicines plus taxes. We calculated step-wise projected incidence and intensity of impoverishment due to medicine purchase. To do this we measured the resources available to individuals as daily per-capita expenditures from the latest National Sample Survey, subtracted daily medication costs, and compared this to 2014 poverty thresholds recommended by an expert group.

**Findings:**

Analysis of cost-effectiveness resulted in five primary prevention drug regimens, created by progressive addition of Aspirin 75 mg, Hydrochlorothiazide 12.5mg, Losartan 25 mg, and Atorvastatin 10 mg or 40mg. Daily cost from steps 1 to 5 increased from Rs. 0·13, Rs. 1.16, Rs. 3.81, Rs. 10.07, to Rs. 28.85. At baseline, 31% of rural and 27% percent of urban Indian population are poor at the designated poverty thresholds. The Rs. 28.85 regimen would be unaffordable to 81% and 58% of rural and urban people. A secondary prevention regimen with aspirin, hydrochlorothiazide, atenolol and atorvastatin could be unaffordable to 81% and 57% rural and urban people respectively. According to our estimates, 17% of the rural 32% of the urban adult population could benefit with these medications, and their out of pocket purchase could impoverish 17 million rural and 10 million urban people in India and increase respective poverty gaps by 2.9%.

**Conclusion:**

Medication costs for cardiovascular disease have the potential to cause financial burden to a significant proportion of people in India. These costs increase the likelihood that patients will forego needed treatment and emphasize the need for programs to reduce the costs of medications for cardiovascular patients in India, including by expansion of prescription drug coverage.

## Background

Health-related expenditures impoverish an estimated 100 million people in low and middle-income countries (LMICs) each year.[1] An estimated 50 million more suffer from catastrophic health expenditures, defined as expenditures of 10% or more of income.[1] As the incidence of non-communicable diseases (NCDs) rises, this is expected to increase,[2, 3] threatening gains in living standards over the past century.[4, 5]

Over 60%, or about 38 million, of all annual global deaths are now due to NCDs, 80% of which occur in LMICs.[2, 3, 6, 7] As populations age, the proportion of NCDs will continue to rise. At 17.5 million deaths per year, cardiovascular deaths are responsible for the greatest proportion of NCD-related deaths,[8] with India projected to have the largest share of these deaths in the coming decade.[9] Furthermore, cardiovascular diseases like coronary artery disease, hypertension, and congestive heart failure are often chronic, requiring long-term treatment and resulting in long periods of disability.

In low and middle income countries, medications constitute the majority of treatment expenditures,[10, 11] so the affordability of treatment is determined largely by the cost of medications. Although researchers have studied the affordability of some medications in LMICs,[12, 13] the financial burden of multiple medicines often prescribed to treat chronic diseases has not been studied. Financial burden due to health expenditures are compounded for lower-income patients, who are affected by NCDs in disproportionately high numbers—contrary to earlier assumptions.[14, 15] The financial burden of poor health can be diminished when most health-related expenditure is covered by insurance. However, over 75% of health expenditure in India is out-of-pocket,[16] with medications comprising 70% of out-of-pocket expenditure.[17, 18] Using aggregated secondary data, we attempt to quantify the financial burden of purchasing cardiovascular medicines in India by measuring the resources available to individuals, subtracting daily medication costs for a medication algorithm to treat ischemic heart disease, and comparing the remaining available resources to established poverty thresholds.

## Data and Methods

Aggressive risk factor reduction remains the cornerstone of primary and secondary prevention of cardiovascular diseases.[19–22] We calculated cost effectiveness for anti-platelet agents, anti-hypertensives and anti-hyperlipidemics as a ratio of daily cost to relative risk reduction for primary prevention of ischemic heart disease. We created step up primary prevention treatment regimens using these three classes of medicines by ordering them based on increasing cost-effectiveness ratios.

### Data

We computed drug prices from the ceiling prices sanctioned by the Government of India’s Department of Pharmaceuticals. Appendix 1 includes the details for this computation. As a comparison, Appendix 1 also discusses prices that we obtained from a web survey of drug prices in India. Supporting information S1 File includes cardiovascular medications included in the National List of Essential Medicines (NLEM) in India. Aggregated household-level expenditure data was obtained from the report of the latest Indian National Sample Survey (NSS)-Round 68 (Modified Mixed Reference Period), 2011–2012, [23] (n = 101,651 households; 59,683 in rural areas and 41,968 in urban areas). The report provides expenditure data for a representative sample of all households in India disaggregated between rural and urban populations. We used poverty thresholds recommended by the Rangarajan Expert Group in July 2014.[24] Thresholds were defined at a per capita daily expenditure of Rs. 46·9 (~74 cents based on market exchange rate, $2·48 based on purchasing power parity) for urban areas and Rs. 32·4 (~ 51 cents based on market exchange rate, $1·70 based on purchasing power parity) for rural areas. In a separate analysis, we also used micro-data from the same NSS-Round 68 to verify our results obtained by using aggregated survey data.

### Methods

We constructed step-up treatment regimens for primary and secondary prevention of ischemic heart disease. Primary prevention regimens are used to prevent the first episode of ischemic heart disease (IHD) from occurring and involve risk factor modification strategies like treating high blood pressure and high cholesterol. Secondary prevention regimens are used after an initial episode of IHD to prevent subsequent episodes. Five primary prevention step-up regimens were created according to diminishing cost-effectiveness. A sixth regimen was also created for secondary prevention. The primary prevention algorithm’s order was: a platelet-inhibitor (aspirin), two anti-hypertensive medicines (hydrochlorothiazide and losartan) and a statin (atorvastatin). The secondary prevention regimen was aspirin, hydrochlorothiazide, atorvastatin, and atenolol (beta blockers like atenolol have additional risk reduction in patients with ischemic heart disease compared to those without, therefore are recommended for use in secondary prevention regimens).[25] Standard recommended drug doses were chosen. Relative risk reduction estimates were obtained from published trials and meta-analyses.[25–29] Risk reduction due to the second anti-hypertensive agent was assumed to be additive.[30] We calculated the cost-effectiveness for each of the medicines used in primary prevention by dividing their daily cost by relative risk reduction. We calculated the financial burden step-wise as the treatment regimen progressed from one to multiple medications, and calculated the effects of this on poverty ratios and poverty gap index for rural and urban households.

We quantified financial burden using aggregated expenditure data from the reports of the NSS-Round 68. We calculated the proportion of people below the poverty threshold (poverty head count ratio) after paying for the medications following our treatment regimens. Since the poverty head count ratio does not capture the intensity of impoverishment, we calculated the poverty gap index (PGI). The PGI[31] measures the depth of poverty, and when micro data are available, it is calculated by summating the income shortfall for every individual below the poverty line and dividing it by the poverty line.

**Fig 1:**
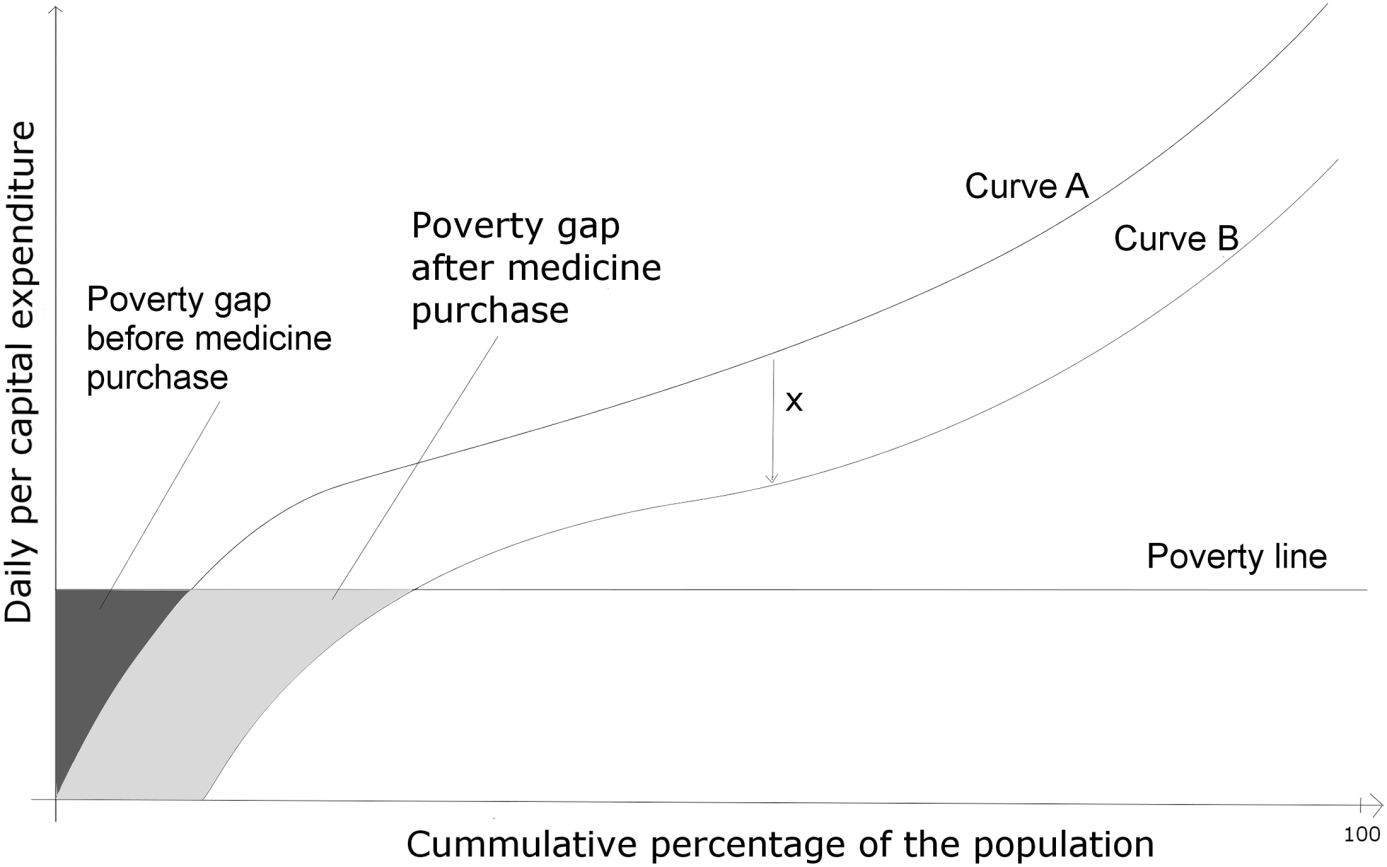
Poverty head count ratio and poverty gap before and after medicine purchase.

As shown in Fig 1, we calculate poverty ratio as the point on the x-axis that corresponds to intersection of the poverty line and the expenditure curve and poverty gap as the area between the poverty line and the expenditure curve (Curve A). We computed the intersection and the area between the poverty lines and the expenditure curves using the mgcv[32] and splancs[33] packages in R (v· 2·15·3). A new expenditure curve (curve B) is plotted for each step after deducting the price of the corresponding regimen and the respective poverty ratios and gaps are calculated. These poverty ratios and gaps, calculated after the purchase of medication represent the gross poverty, and the resulting difference from the baseline poverty ratios and gaps represents the net poverty. This net poverty is the impoverishing effect due to the purchase of the medicine. The gross poverty calculated at each step represents the percentage of people for whom the given regimen is unaffordable.

### Estimation of people needing cardiovascular medicine

The poverty estimations thus far essentially give the percentage of people out of the total population, and not the diseased population, who are at risk of suffering from financial burden if they had to buy these medicines out of pocket. While such estimations are able to tell us the proportion of people who may be at the risk of financial burden, the estimations remain hypothetical because not every one will require these medicines. We use estimates of the short term risk of cardiovascular diseases from the Indian Surveillance Sentinel Study[34] among the industrial population to estimate the number of people requiring cardiovascular medicines in the general population. We assume that people with a high short-term risk (10 year risk of CVD greater than 10%) of cardiovascular disease require these regimens (people with high short-term risk of cardiovascular diseases benefit from primary prevention with Aspirin, and potentially anti-hypertensives and anti-hyperlipidemics).[21, 35] We use estimates of prevalence of hypertension, hyperlipidemia and the co-occurrence of these conditions in India to calculate the percentage of people who may benefit from these regimens. We then use these estimates as the denominator to estimate the number of people that could be financially burdened or impoverished by these medicines, by applying the estimates of financial burden obtained in the first part of our calculations. To further explore the variation in our estimates of population at risk of cardiovascular diseases, we perform a univariate sensitivity analysis, by sequentially increasing or decreasing the variables used to estimate the percentage of people in each regimen, by 10%. While calculating poverty figures, we assume that expenditure, and therefore available resource distribution, is the same in these groups as in the general population.

### Aggregate data versus micro data

When micro-data are available financial burden can be accurately calculated using the impoverishment method as outlined by O’Donnell et al.[36] Niens et al.[37] attempted to calculate financial burden using aggregate data, however their method assumes that income within a decile is equal for everyone. This can be a source of considerable error. Our method avoids such averaging, because we interpolate the aggregate expenditure data points by using monotonic cubic splines (function “splinefun” with option “hyman” in R), in order to create accurate expenditure curves. We verified results that we obtained by analyzing aggregated expenditure data by comparing them with results that we obtained by analyzing micro data from the same sample survey.

### Findings

Table 1 presents aggregate Monthly Per Capita Expenditures (MPCE) for rural and urban India. Commonly available cardiovascular medicines in India with their prices are presented in Table 2. Aspirin, hydrochlorothiazide, losartan and atorvastatin were found to be the most cost effective medicines for primary prevention in the NLEM (Table 3). Five primary prevention regimens were created with these medicines. Table 4 presents total costs for the regimens including aspirin alone, aspirin and one anti-hypertensive, aspirin and two anti-hypertensives, and aspirin and two anti-hypertensives with a statin. A 4 drug regimen for primary prevention with aspirin 75mg, hydrochlorothiazide 12.5 mg, losartan 25 mg and atorvastatin 40mg (step 5) was found to cost Rs. 28·85.

**Table 1:** Per capita expenditure distribution for rural and urban India according to cumulative percentage of the population.

Cumulative % of the population	Monthly Per Capita Expenditure (Rs.)	
	Rural	Urban
0–5	616	827
5–10	710	983
10–20	845	1239
20–30	963	1490
30–40	1075	1757
40–50	1198	2019
50–60	1341	2349
60–70	1522	2771
70–80	1793	3390
80–90	2296	4610
90–95	2886	6383
95–100	—	—

Note: Source: Key Indicators of Household Sample Survey in India, NSS Round 68, June 2013. MPCE figures represent the upper bound of expenditure for the respective percentile bracket. 1 US$ = 63.45 Indian Rs.

**Table 2:** Common drugs for cardiovascular disease prevention in the national essential medicines list and their prices.

Therapeutic class	Medicine	Daily cost in Rupees
		(incl. tax)
Anti-platelet	Aspirin 75mg	0.13
Anti-hyperlipidemic	Atorvastatin 5 mg	4.04
	Atorvastatin 10 mg	6.26
Beta blocker	Atenolol 50 mg	2.19
	Atenolol 100mg	3.94
	Metoprolol 25 mg	11.86
ACE/ ARB	Losartan 25 mg	2.65
	Losartan 50 mg	4.55
	Enalapril 2.5mg	3.77
	Enalapril 5 mg	6.27
Anti-hypertensive	Hydrochlorothiazide 12.5mg	1.03
	Amlodipine 5 mg	3.24
	Nifedipine SR 30mg	3.52

Note: Drugs taken from the National List of Essential Medicines. Prices are as published in the Drug Price Control Order (DPCO) 2013. Prices include a 0% excise duty and a 6% sales tax (Value Added Tax).

**Table 3:** Cost-effectiveness of cardiovascular medicines for primary prevention of Ischemic Heart Disease.

Therapeutic class	Medicine	Daily cost (incl. tax)	Primary prevention relative risk (RR) of IHD (95% CI)	Primary prevention cost/ RR reduction
Anti-platelet	Aspirin 75mg	0.13	0.68 (0.60–0.77)	0.40
Anti-hypertensive	Hydrochlorothiazide 12.5mg	1.03	0.75 (0.63–0.87)	4.12
	Losartan 25 mg	2.65	0.66 (0.60–0.77)	4.41
Anti-hyperlipidemic	Atorvastatin 10 mg	6.26	0.73 (0.67–0.80)	8.57

Note: Relative risk reduction is for Ischemic Heart Disease. Risk reduction estimates of ischemic heart disease (IHD) obtained from published trials. Risk reduction for second anti-hypertensive agent assumed to be additive. Primary prevention refers to prevention of the first episode of IHD while secondary prevention refers to the prevention of subsequent episodes of IHD after the first one. No cost-effectiveness information is provided for atenolol because it has not been used in our primary prevention regimens.

**Table 4:** Cardiovascular disease primary and secondary prevention step-up regimens with their prices.

Aspirin 75mg	Hydrochlorothiazide 12.5mg	Atenolol 50mg	Atorvastatin 40mg	**Secondary prevention: STEP 6**	Rs. 28.39
Aspirin 75mg	Hydrochlorothiazide 12.5mg	Losartan 25 mg	Atorvastatin 40mg	**Primary prevention: STEP 5**	Rs. 28.85
Aspirin 75mg	Hydrochlorothiazide 12.5mg	Losartan 25 mg	Atorvastatin 10mg	**Primary prevention: STEP 4**	Rs. 10.07
Aspirin 75mg	Hydrochlorothiazide 12.5mg	Losartan 25 mg	**Primary prevention: STEP 3**		Rs. 3.81
Aspirin 75mg	Hydrochlorothiazide 12.5mg	**Primary prevention: STEP 2**			Rs. 1.16
Aspirin 75mg	**Primary prevention: STEP 1**				Rs. 0.13

Note: Primary prevention regimens created based on increasing cost-effectiveness ratios. Secondary prevention regimen uses atenolol, a beta-blocker because they offer additional risk reduction in secondary prevention. All medicines are from the National List of Essential Medicines (Supporting Information S1 File). Doses are as recommended in standard pharmacopoeia. Drug prices as published in the Drug Price Control Order (DPCO) 2013. Primary prevention regimens are used to prevent the first episode of ischemic heart disease by modifying risk factors like hypertension and hyperlipidemia, while secondary prevention regimens are used to prevent subsequent episodes of ischemic heart disease after the first one. Beta blockers like Atenolol, used in secondary prevention regimen (step 6) are not recommended for use in primary prevention.

Treatment cost for a 4 drug secondary prevention regimen (step 6) with aspirin 75mg, hydrochlorothiazide 12·5 mg, atenolol 50 mg and atorvastatin 40mg was found to be Rs. 28·39.

**Fig 2:**
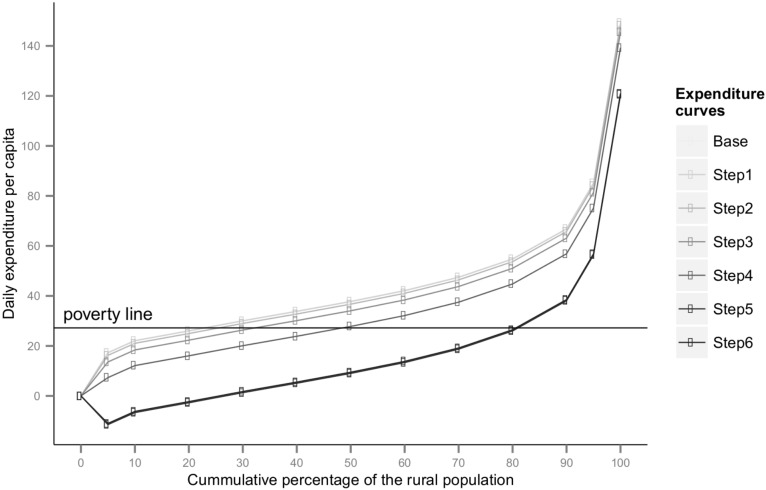
Expenditure curves for rural India before and after medicine purchase.

**Fig 3:**
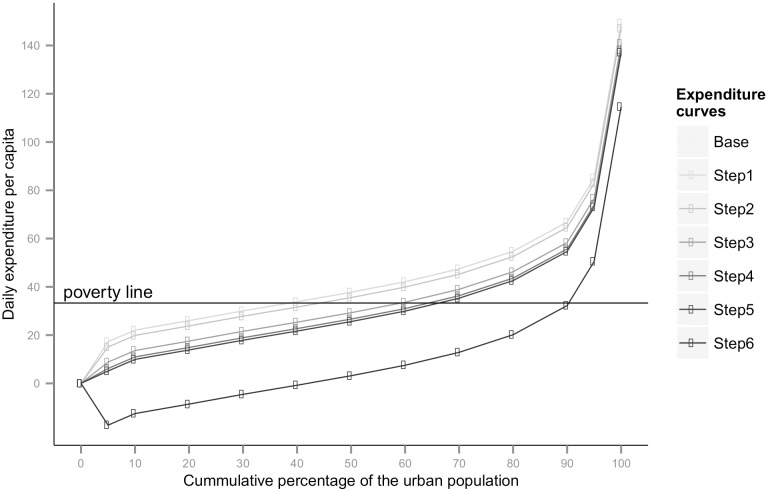
Expenditure curves for urban India before and after medicine purchase.

At the Rs. 32.4 poverty threshold for rural India, 31% of the rural population is poor. At the Rs. 46.9 poverty threshold for urban India, 27% of the urban population is poor. The baseline poverty gap indices are 8% and 6% for rural and urban India respectively. These baseline poverty figures that we calculated were the same as the figures calculated by the government appointed Rangarajan Expert Group. To the population below the poverty line any health expenditure may be assumed to be unaffordable. A primary prevention regimen of an aspirin and an anti-hypertensive (hydrochlorothiazide 12·5 mg—step 2) increase and gross poverty ratios to 34% and 28% of rural and urban people respectively and therefore be unaffordable to this proportion of the population, if they were to require these medicines. This regimen could impoverish an additional 3% rural and 1% in urban population (Figs 2 and 3) compared to the baseline. The corresponding increase in PGI would be 0·54% in rural areas and 0·25% in urban areas. These calculations of poverty ratios and gaps are hypothetical for reason outlined in the methods section.

Adding a second antihypertensive (losartan 25 mg—step 3) to the base regimen could increase gross poverty ratios to 40% and 31% in rural and urban India respectively; gross poverty-gaps indices could increase to 12% in rural areas and 10% in urban areas. The step 4 regimen, with the addition of atorvastatin 10 mg to the step 3 regimen, could be unaffordable to 45% of the rural population and 38% of the urban population. A regimen using 40mg dose of atorvastatin (step 5) could be unaffordable to 81% people in rural and 58% of people in urban India (Table 5) and would cause the poverty gap indices to increase to 60% and 29% respectively. This regimen could impoverish 50% of rural and 31% of urban people in addition to those already poor at baseline. A secondary prevention regimen using aspirin 75 mg, atenolol 50 mg, hydrochlorothiazide 12·5mg and atorvastatin 40mg (step 6) could be unaffordable to a similar percentage of people, impoverishing 50% more rural and 31% more urban people. The corresponding PGI increase would be 52% and 23%.

**Table 5:** Poverty ratio and poverty gap index for rural and urban India before and after medication purchase, calculated by using aggregate data.

Regimen	Rural		Urban	
	Poverty ratio % (Increase from baseline %)	Poverty gap index % (Increase from baseline %)	Poverty ratio % (Increase from baseline %)	Poverty gap index % (Increase from baseline %)
Baseline poverty	30.80	7.57	26.69	6.22
Primary prev. Step 1	31.15 (0.35)	7.63 (0.06)	26.84 (0.15)	6.25 (0.03)
Primary prev. Step 2	33.91 (3.11)	8.11 (0.54)	28.07 (1.38)	6.47 (0.25)
Primary prev. Step 3	40.91 (10.11)	11.69 (4.13)	31.17 (4.48)	9.82 (3.60)
Primary prev. Step 4	55.32 (24.52)	19.11 (11.54)	38.20 (11.51)	11.82 (5.60)
Primary prev. Step 5	80.88 (50.08)	60.09 (52.52)	57.68 (30.99)	29.01 (22.79)
Secondary prev. Step 6	80.16 (49.81)	59.32 (51.95)	57.26 (30.57)	28.77 (22.54)

Notes: Increase from baseline is in terms of poverty ratio or poverty gap percentage points. Poverty ratio and gap index indicates gross poverty while increase from baseline indicates net poverty ratio and gap index. Poverty figures are expressed as a percentage of the entire population and the not just the population with cardiovascular disease.

Our results from the aggregate data closely matched the results that we obtained by analyzing the micro-data. A comparison is given in Table 6 and the detailed results from our analysis of the micro-data are given in supporting information File 1.

**Table 6:** Rural and Urban poverty ratio for India calculated with aggregate and micro data and the difference between the two, before and after medicine purchase.

	Rural			Urban		
	Aggregate- data %	Micro-data %	Difference %	Aggregate-data %	Micro-data %	Difference %
Baseline	30.8	30.8	0	26.69	26.76	-0.07
Regimen 1	31.15	31.22	-0.07	26.84	26.9	-0.06
Regimen 2	33.91	34.05	-0.14	28.07	28.04	0.03
Regimen 3	40.91	41.05	-0.14	31.17	31.14	0.03
Regimen 4	55.32	55.79	-0.47	38.2	38.14	0.06
Regimen 5	80.88	81.18	-0.3	57.68	57.75	-0.07
Regimen 6	80.16	80.79	-0.63	57.26	57.33	-0.07

Note: Difference is the number of poverty ratio percentage-points difference between the poverty ratios calculated using aggregate and micro-data. Poverty ratios are gross figures after the purchase of the corresponding regimen. Table with detailed figures for calculations using micro-data are given in supporting information S1 File

### Estimates of people needing cardiovascular medicines

**Fig 4:**
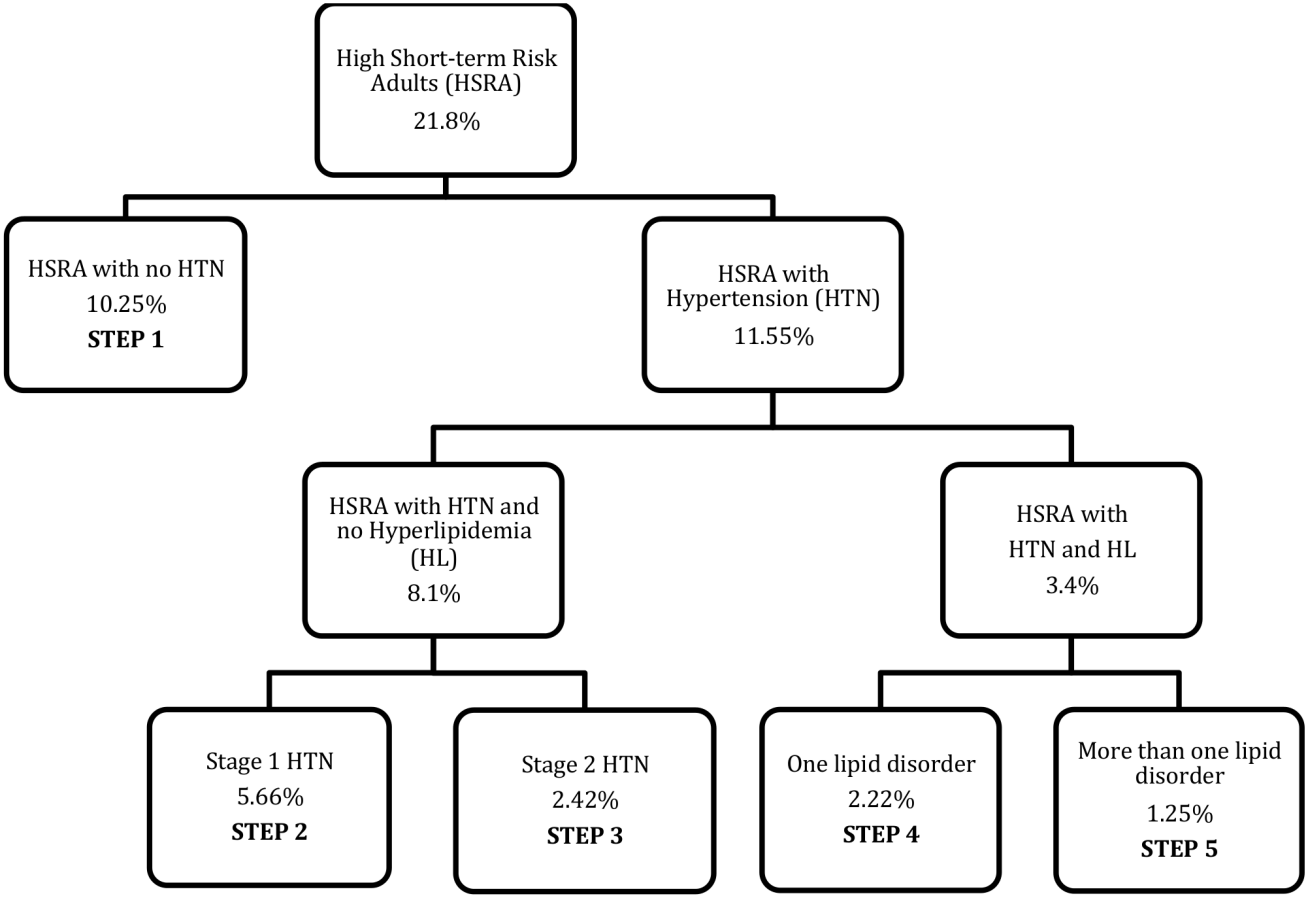
Schematic representation of the percentage of urban adults in primary prevention regimens. Note: Our assumptions, based on clinical recommendations, are as follows: 1. All adults with high short term risk of CVD require at least an aspirin. All adults with stage 1 hypertension (BP 140-159/90-99) require treatment with 1 anti-hypertensive medication and an aspirin. All adults with stage 2 hypertension require 2 anti-hypertensive medications and aspirin. We also assume that all adults with a single lipid disorder require treatment with low dose statin and all adults with more than one lipid disorder require treatment with high dose statin. The following estimates are used in our calculations, and they are based on published studies: percentage of urban adults with high short-term risk is 21.8%. Among these, percentage of adults with hypertension is 53%. Percentage of hypertensive adults with stage 1 hypertension is 70%. Percentage of hypertensive adults with both hypertension and hyperlipidemia is 30%. Among these hypertensive-hyperlipidemic adults, percentage of adults with single lipid disorder is 64% and more than one lipid disorder is 36%. The same percentages apply for rural adults, except that the percentage of rural adults with high short-term risk of cardiovascular disease is 12%.

In Appendix 2, we explain how we used findings from the Indian Sentinel Survey Study to estimate the number of people needing cardiovascular medicines. In short this was done by breaking down the percentage of people with high short term risk of CVD into appropriate regimen categories based on their hypertension or hyperlipidemia status. This breakdown for urban India is illustrated in a schematic in Fig 4. Breakdowns for rural India can be done in a similar fashion. Based on our findings, the percentage of rural population requiring a primary prevention regimen is 12%, out of which 5.64% requires a Step 1 regimen, 3.12% requires a Step 2 regimen, 1.34% requires a Step 3, 1.22% requires a Step 4 and 0.69% requires a Step 5 respectively. In urban areas, the percentage of urban population requiring a primary prevention regimen is 21.8%. Out of this 10.25% requires Step 1, 5.66% requires Step 2, 2.43% requires Step 3, 2.22% requires a Step 4 and 1.25% requires a Step 5 regimen. Meta analysis have put estimates of coronary heart disease prevalence at 10.5% of the adult population in urban areas and 4.5% in the rural areas of India.[38] These people require the Step 6 regimen for secondary prevention of CVD.

Our finding show that as much as 17% (93 m.) of the rural and 32% (76 m.) of the urban adult population may benefit from cardiovascular medicines (Table 7). If all of them were to buy cardiovascular medicines out of pocket, 4.05% of the urban adult population and 3.14% of the rural adult population could be impoverished due to the purchase. This could mean that 17 million people in rural areas and 10 million people in urban areas could be impoverished if all the people who would benefit from these medicines bought them out of pocket. Poverty gaps could increase by as much as 2.91% in rural areas and 2.88% in urban areas (Table 7).

**Table 7:** Estimates of the number of people that could benefit from cardiovascular medicines and the resulting number of people financially burdened or impoverished due to medication purchase.

Regimen	Adult population on the particular regimen %^#^ ^$^	Unaffordable % ^¶^	Impoverished %^¶^	Adult population Impoverished due to regimen % ^&^	Increase in poverty gap due to the regimen ^¶^	Actual increase in poverty gap due to the regimen use ^ε^	Population burdened in millions ^§^	Population impoverished in millions ^ϕ^
Rural India								
Step 1	5.64	31.15	0.35	0.02	0.06	0.00	9.57	0.11
Step 2	3.12	33.91	3.11	0.09	0.54	0.02	5.76	0.53
Step 3	1.34	40.91	10.11	0.15	4.13	0.06	2.98	0.74
Step 4	1.22	55.32	24.52	0.18	11.54	0.14	3.68	1.63
Step 5	0.69	80.88	50.08	0.38	52.52	0.36	3.03	1.87
Step 6	4.50	80.16	49.81	2.24	51.95	2.34	19.66	12.22
Rural Total^~^	16.50			3.07		2.91	44.68	17.09
Urban India								
Step 1	10.25	26.84	0.15	0.02	0.03	0.00	6.55	0.04
Step 2	5.66	28.07	1.38	0.10	0.25	0.01	3.78	0.19
Step 3	2.43	31.17	4.48	0.16	3.60	0.09	1.80	0.26
Step 4	2.22	38.20	11.51	0.21	5.60	0.12	2.02	0.61
Step 5	1.25	57.68	30.99	0.56	22.79	0.28	1.71	0.92
Step 6	10.50	57.26	30.57	3.21	22.54	2.37	14.31	7.64
Urban Total^~^	32.30			4.25		2.88	30.17	9.65

^$^ Estimates of the adult population requiring primary prevention regimen (Step 1–5) are based on the surveys of cardiovascular disease risk in a sentinel survey of industrial workers, and surveys of prevalence of hypertension and hyperlipidemia (see Fig 5). Estimates of the adult population requiring secondary prevention regimen (step 6) are obtained from a meta-analysis of surveys of cardiovascular disease prevalence in India. Population on any given regimen is the population that benefits from that regimen

^#^ Numbers are percent of the rural or the urban adult population respectively…

^¶^ Figures are obtained from Table 5.

^&^ Assuming everyone bought their medicine out of pocket.

^ε^ Figure obtained by multiplying the second column with the sixth column.

^§^ Figure obtained by multiplying the second and third column with the respective adult population.

^ϕ^ Figure obtained by multiplying the fifth column with the respective adult population.

^~^ Totals are rural and urban totals respectively.

Adult population (above 20 years) in rural India is 545 million. Adult population in urban India is 238 million. All figures are rounded to 2 decimal places.

**Fig 5:**
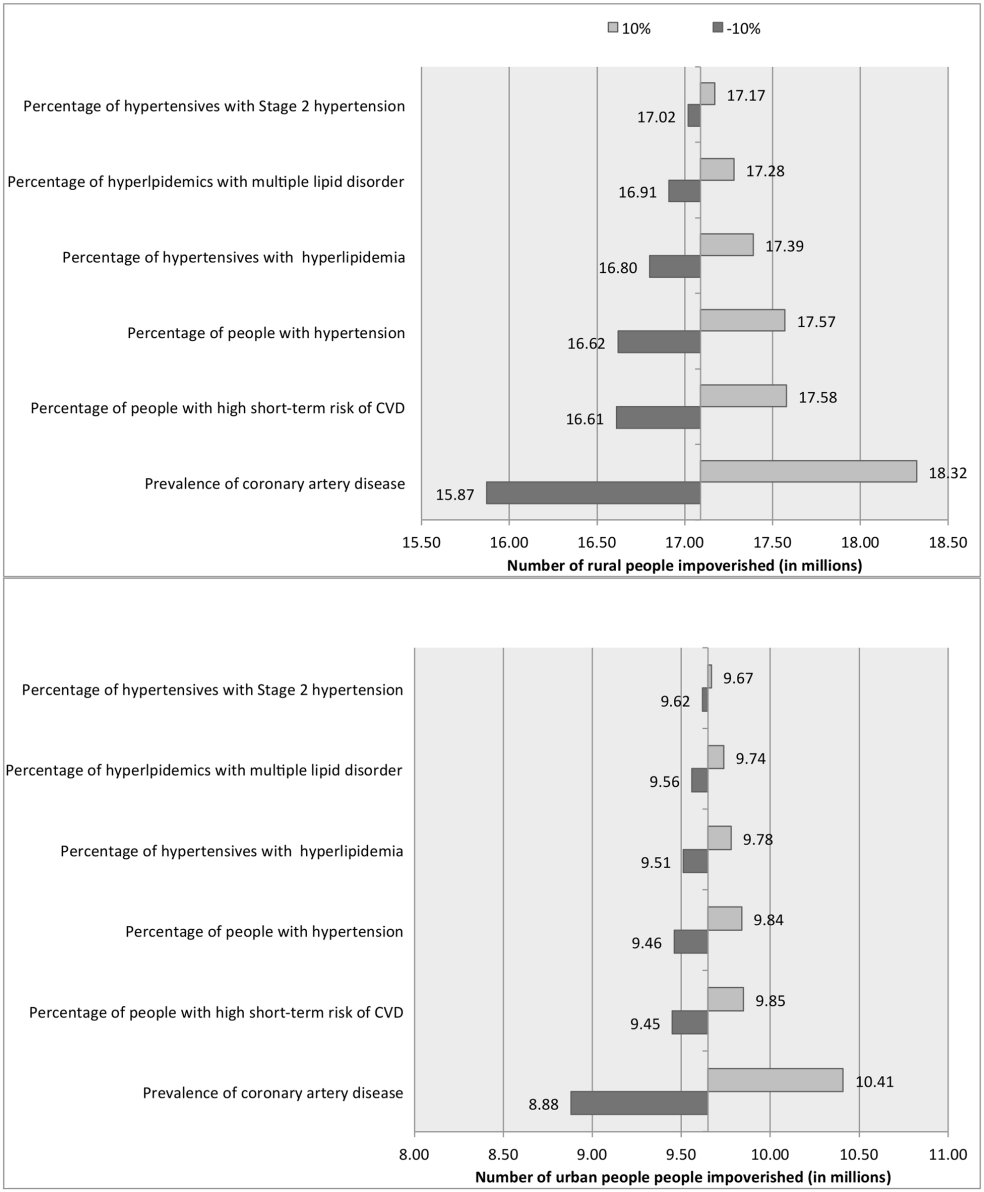
Univariate sensitivity analysis showing the change in the number of rural and urban adults impoverished with a 10% change in each of the variables that determine the potential user population.

Our sensitivity analysis reveals that a 10% change in one of the variables used in estimating the number of people requiring these regimens could change the estimates of the number of people facing impoverishment in the range of 15.87–18.32 million in rural India (Fig 5), from a baseline estimate of 17.09 million and in the range of 8.88–10.41 in urban India (Fig 5) from a baseline estimate of 9.65 million. The greatest change is brought by the change in the number of people who require the step 6 regimen, because the percentage of people who require this regimens is large and also because these regimens are expensive.

## Discussion

The results of our study show that the more expensive cardiovascular medicine regimens could be unaffordable to as much as 81% of the rural and 58% of the urban population in India if they wanted to purchase these medicines out of pocket. If all of the 93 million of the rural and 76 million of the urban adult population who may benefit from cardiovascular medicines were to buy these medications out of pocket, as many as 45 million rural and 30 million urban Indians could be financially burdened with impoverishment of 17 million rural and 10 million urban people. These figures represent an increase in baseline poverty levels by 2 percentage points for rural India and 3.2 percentage points for urban India. Poverty gaps could increase by 2.91 percentage points in rural India (from a baseline of about 7.6%) and 2.88 percentage points in urban India (from a baseline of about 6.2%). As a percentage of baseline poverty levels, the poverty ratios could increase by about 6% and 11% for rural and urban areas respectively. However the poverty gap indices could increase by about 38% of baseline for rural India and by about 46% for urban India. This shows that the financial burden posed by cardiovascular medication purchase could be significant and much more severe than that represented by the increase in the number of people pushed below the poverty line alone. If 75% of these people were to buy cardiovascular medicines out of pocket, as is the current share of out of pocket health expenditures in India, as many as 34 million rural Indians and 22 million urban Indians could find these medicines unaffordable with resulting impoverishment of 13 million rural Indians and 7 million urban Indians.

Several studies[17, 39, 40] have attempted to study the financial burden resulting from health expenditures using reported health expenditure data. Some studies[13, 37] have attempted to study the financial burden of a single medication. van Doorslaer et al.[17] used retrospective household survey data of health expenditures and found that 4% of people in India are impoverished at the $1·08 poverty line due to health expenditures with a corresponding PGI increase of 1%. According to van Doorslaer et al., 78 million additional people will be below the $1 poverty line in the 11 low-income countries of Asia if health related expenditures are deducted from household resources. Niens et al.[13] estimated that 40% of the 775 million people in LMIC they studied are not able to afford essential medicines. The Niens estimates are high because, like our estimates, they are prospective estimates of financial burden. However they don’t account for the number of people who may actually need these medicines. With our estimation of people who may actually need these medicines, we are able to estimate the financial burden more accurately.

Multi-drug regimens for chronic diseases pose a significant burden on the population. Since chronic diseases like CVD are rarely treated with just one drug, our analysis offers a more realistic estimate of the actual financial burden due to these drugs. Furthermore, the more dramatic increase in poverty gap index with the most expensive regimen compared to poverty ratio suggests that the actual financial burden due to these medicines is much greater than that demonstrated by the poverty ratios alone. Although the government of India has declared essential medicines free within the public health system, availability has often been reported to be anywhere between 0 to 30%.[41] Health insurance schemes like the Rajeev Swasthya Bima Yojana (RSBY) and AarogyaShri are now available for some people in India, but they mostly cover hospital charges and do not provide long-term prescription coverage.[42] As a result, 70% of out-of-pocket health expenditures in India are for purchasing drugs.

Our methodology using aggregated data was consistently able to match poverty ratios from micro data to within half a percentage-point (Table 6) and poverty gap index within 0.5–2.5% percentage-points (supporting information S1 File). We have estimated prospective financial burden due to expenditures for cardiovascular medications, an approach that offers some advantages and disadvantages over using reported health expenditure data. Health expenditures may be discretionary. More than half of chronic disease expenditure among poor households is reportedly borrowed,[43] thereby inflating their ability to afford medicines. Some households may decide to forgo care. Reported expenditure data is also likely to suffer from recall bias. Prospective estimation however, will not be able to reflect the reduced ability to pay due to indirect costs (inability to work, lost productivity, and lost income) of an illness. And unless attempts are made to estimate the number of people who actually need these medications, prospective estimation is hypothetical and only gives the number of people potentially at risk of financial burden.

Our study has some important limitations. Our use of government sanctioned ceiling prices (which are based on the average of the prices of the top 10 selling generics) to calculate the price of the regimens could overestimate what patients, especially those who are price conscious, are likely to pay for these regimens because cheaper options are available in the market as shown by our web survey, pushing poverty estimates down. However the prices the average consumer is likely to pay is still better represented by the ceiling prices because ceiling prices are also reflective of the market share. Second, we have not taken into account health related expenditures collected as part of the NSS. Third, our study does not account for the people who may be receiving free medicines from the public health system or prescription coverage from their health insurance where available. Their number however is likely to be relatively low, for reasons explained above. We have also not been able to account for poverty mitigating effects of various subsidy schemes for people below the poverty line and the effect these schemes may have in overall resource availability and financial burden. Our study is also unable to account for the indirect poverty due to a loss of income caused by cardiovascular diseases, or the incomes gains if any, due to treating cardiovascular diseases. We also assume that per capita expenditure distribution is similar in each of the treatment groups. Although 75% of medication expenditure may be out of pocket, if insurance schemes and free health services are able to selectively target poorer people, this would significantly reduce the number of people that are burdened by health expenditures.

Our estimates of the number of people needing cardiovascular medicines also make several assumptions. First, the Indian sentinel survey study from which we base our calculations used the ATP III formula to calculate the cardiovascular risk, a formula that was based out of the Framingham study in the US and has not been calibrated for India. This formula heavily depends on the age of the person, has been known to be inaccurate in the extremes of values. Indian populations have known to have higher cardiovascular risk at a younger age and this may result in underestimation of short term risk of CVD. Applying findings from RCTs done primarily in the west to the Indian population may also be a source or erroneous estimates. The accuracy of our estimates are also contingent upon the accuracy of the other published estimates of the categories of hypertension, hyperlipidemia and the co-occurrence of hypertension-hyperlipidemia that we base our calculations on. According to some surveys the prevalence of hypertension and hyperlipidemia may be higher than estimates from the Indian Sentinel Survey Study.[44, 45] Our calculations also do not offer estimates of high short term risk patients with hyperlipidemia without hypertension, even as this population could benefit from primary prevention as the results of the HOPE-3 trials show.[46] Our calculations are based on the assumption that everyone with a more than 10% risk for CVD will benefit from primary prevention regimens, an assumption that is debatable, especially given that these recommendations are not able to account for personal preferences of patients. If we were to increase this risk threshold, it would significantly reduce our estimates. Finally, our sensitivity analysis is limited because it is not probabilistic.

## Conclusion

We found that cardiovascular medications could be unaffordable to a significant proportion of patients in India, and can pose a financial burden with resulting impoverishment. With the increasing incidence of cardiovascular diseases in LMICs, medication-related costs could potentially reverse the gains made over recent years in poverty alleviation in many of these countries. Furthermore, poorer patients may forgo care, with potentially devastating consequences for public health. This suggests a need for strategies to mitigate the burden resulting from medication-related expenditures.

## APPENDIX 1: Drug Prices in India and Web Survey of Drug Prices

The department published its Drug Price Control Order (DPCO) 2013[47] in May 2013, which includes statutorily binding ceiling prices for 348 essential medications (652 formulations) in the National List of Essential Medicines (NLEM). Supporting information S1 File includes cardiovascular medications included in the NLEM. Manufacturers are obligated to label units with the Maximum Retail Sales Price (MRSP), a sum of the ceiling price for each medicine plus the sales tax and the excise duty. We computed the MRSP based on the prevalent 0% excise duty and 6% sales tax (VAT) for medicines.[48] For medicines scheduled in the NLEM, the MRSP is the maximum price retailers are permitted to charge, and is usually what customers pay. While drug prices are sometimes surveyed using the WHO/Health Action International methodology of randomly sampling end-user prices at public, private and nonprofit pharmacies,[49] such surveys are limited to a few medications and often several years old, and therefore may not reflect current market prices.

For each of the medicines in our study, we did a web based survey of drug company published maximum retail sales prices (they are statutorily obligated to declare such prices) in India using a price comparison search engine.[50] We found prices from 23 manufacturers for Aspirin 75mg, 10 for Hydrochlorothiazide 12·5mg, 37 for Atenolol 50mg, 53 for Atorvastatin 10 mg and 8 for Atorvastatin 40mg. We also obtained prices for combination pills (poly-pills) for atorvastatin 10mg/aspirin75 mg and losartan 50mg/hydrochlorothiazide 12·5mg from the same source to compare if the use of poly-pills made a regimen less costly.

From random sampling of the web based survey prices, the mean price of a primary prevention regimen including aspirin 75mg, hydrochlorothiazide 12·5mg, losartan 25mg, and atorvastatin 40mg was Rs. 23·63 (95% CI 23.58–23.85; cheapest 19.59, most expensive 32.78). Based on the web survey, mean price for aspirin 75mg and atorvastatin 10mg combination pill was found to be Rs. 6·47 compared to Rs. 6·49 when buying them as individual pills. Similarly mean price for a combination of losartan 25mg and hydrochlorothiazide 12·5 was Rs. 5·02 and Rs. 3·33 when bought as individual pills.

Although India produces as much as 20% of the world’s generic medicines, our study illustrates that essential generic medicines can be out of the reach of many people who need them. In a high-income country like the Unites States, a generic prescription similar to the most expensive regimen in our study is available for a total cost of as little as $10 dollars per month (about an hour-and-a-half’s earnings at minimum wage) even when paid out of pocket, compared to almost the entire month’s income for a low income patient in India. In the absence of assistance for medicine purchase, the situation in other LMIC could be worse because while they often have similar income distribution as India, equivalent generic medicines may not be as readily available and the brand name version may be more expensive. For example, Pfizer sells it’s brand name Atorvastatin 10 mg tablet for $1.31 per tablet in China, $1.44 in Indonesia and $0.72 in Philippines, compared to a ceiling price of $0.10 for Atorvastatin 10 mg tablet in India.[51] Pfizer’s brand name Atorvastatin is not available in India. And although poly-pills have often been suggested as a means of reducing pill burden as well as reducing the overall cost of multi drug regimens, our web survey reveals that in India poly-pills are no cheaper than the individual pills that they try to replace.

## APPENDIX 2: Estimation of People Needing Cardiovascular Medicines in India

According to the Indian Sentinel Survey Study[34], 21.8% of the urban and 12% of the peri-urban (rural) industry workers and their families between ages 20 to 69 had a high short-term risk of cardiovascular disease. In order to estimate the likely number of people who may need primary prevention regimens, we assumed that the risk levels were the same in the general population above the age of 20, including people above the age of 69 (the risk is likely to be higher but this age group represents less than 1.5% of India’s population). Primary prevention regimens, with at least an aspirin (Step 1), are recommended for all people with a high short term risk for CHD.[21, 35] Furthermore according to the same survey 53% of the people with high short term risk had hypertension as well, therefore requiring either a Step 2 or Step 3 regimen (these are regimens for people with hypertension). However, as much as 30% of people with hypertension have been found to have hyperlipidemia,[52] thereby requiring a regimen with a statin as well (step 4 or 5). We use estimates of stage 1 (BP 140-159/90-99) and stage 2 hypertension to determine people who need step 2 (with 1 anti-hypertensive) or step 3 (with 2 anti-hypertensive) regimens. 70% of hypertensives in India are estimates to have stage 1 hypertension.[53] We base our estimates of people who need the Step 4 regimen (low dose statin) on the percentage of people with a single lipid disorder and the high dose on more than one lipid disorder. According to one survey, 64% of people with hyperlipidemia in India were found to have a single lipid disorder.[44] Except for the estimates of adults with high short-term risk of cardiovascular diseases, for the sake of simplicity in our assumptions, we use the same figures for hyperlipidemia and hypertension prevalence and co-occurrence to calculate the number of people in each regimen in rural as well as urban India.

## Supporting Information

S1 File (DOCX)
