# Supplementary material for: Financial Burden and Impoverishment Due to Cardiovascular Medications in Low and Middle Income Countries: An Illustration from India
Source: PLoS One. 2016 May 9;11(5):e0155293. doi: 10.1371/journal.pone.0155293 (PMC4861328; doi:10.1371/journal.pone.0155293)
Supplement: S1 File — (DOCX) [file pone.0155293.s001.docx]

| Supporting Information File 1  **Cardiovascular medicines in the National List of Essential Medicines^*^, published by the Department of Pharmaceuticals, India** | | | |
| --- | --- | --- | --- |
|  |  |  |  |
| **Cardiovascular medicines** | | | |
| **Medicines** | **Category^#^** | **Route of Administration/ Dosage form** | **Strengths** |
|  |  |  |  |
| **1: Antianginal medicines** | | | |
| Acetyl salicylic acid | P,S,T | Tablets | 75mg, 100mg |
|  |  |  | 350 mg dispersible |
| Diltiazem | S, T | Tablets | 30 mg, 60 mg |
| Glyceryl | P,S,T | Sublingual Tablets | 0.5 mg |
| Trinitrate |  | Injection | 5mg/ml |
| Isosorbide | P,S,T | Tablets | 10 mg |
| Mononitrate/ |  |  | 20 mg |
| Dinitrate |  |  |  |
| Metoprolol | P,S,T | Tablets | 25 mg, 50 mg |
|  |  | Injection | 1mg/ml |
| Clopidogrel | T | Tablets | 75 mg |
| **2: Antiarrhythmic medicines** | | | |
| Adenosine | S,T | Injection | 3 mg/ml |
| Amiodarone | S, T | Tablets | 100 mg, 200 mg |
|  |  | Injection | 50 mg/ml (3 ml ampoule) |
| Diltiazem | S, T | Tablets | 30 mg, 60 mg |
|  | T | Injection | 5 mg/ml |
| Esmolol | T | Injection | 10 mg/ml |
| Lignocaine Hydrochloride | S, T | Injection | 1%, 2% |
|  |  |  |  |
| Procainamide Hydrochloride | T | Tablets | 250 mg |
|  |  | Injection | 100 mg/ml |
| Verapamil | S, T | Tablets | 40 mg, 80 mg |
|  |  | Injection | 2.5 mg/ml |
| **3: Antihypertensive medicines** | | | |
| Amlodipine | P,S,T | Tablets | 2.5 mg |
|  |  |  | 5 mg |
| Atenolol | P,S,T | Tablets | 50 mg |
|  |  |  | 100 mg |
| Enalapril | P,S,T | Tablets | 2.5 mg, 5mg |
| Maleate | T | Injection | 1.25 mg/ml |
| Losartan Potassium | S, T | Tablets | 25 mg |
|  |  |  | 50 mg |
| Methyldopa | P,S, T | Tablets | 250 mg |
| Nifedipine | S, T | Capsules | 5 mg, 10mg |
|  |  | Tablets | 10 mg, 20 mg |
|  |  | Sustained release tablets or capsules | 10 mg, 20 mg |
| Sodium | T | Injection | 50 mg/5 ml |
| Nitroprusside |  |  |  |
| Hydrochlorthiazide | P,S,T | Tablets | 12.5 mg |
|  |  |  | 25 mg |
| **4: Medicines used in heart failure** | | | |
| Digoxin | S, T | Tablets | 0.25 mg |
|  |  | Injection | 0.25 mg/ml |
|  |  | Elixir | 0.05 mg/ml |
| Dobutamine | S, T | Injection | 50 mg/ml |
| Dopamine | S,T | Injection | 40 mg/ml |
| Hydrochloride |  |  |  |
| **5: Antithrombotic/ thrombolytic medicines** | | | |
| Clopidogrel | T | Tablets | 75mg |
| Acetyl salicylic acid | P,S,T | Tablets | 75 mg |
|  |  |  | 100 mg |
|  |  |  | 350 mg dispersible |
| Heparin Sodium | S, T | Injection | 1000 IU/ml |
|  |  |  | 5000 IU/ml |
| Streptokinase | S, T | Injection | 750,000 IU/ml |
|  |  |  | 1500,000 IU/ml |
| Urokinase | T | Injection | 500,000 IU/ml |
|  |  |  | 1000,000 IU/ml |
| **6 Anti-Hyperlipidemic Medicines** | | | |
| Atorvastatin | P,S,T | Tablets | 5 mg, 10 mg |
| Notes: ^*^As published in the Drug Price Control Order 2013. **^#^** Health facility category P: Primary S: Secondary T: Tertiary | | | |
|  | | | |

| **Poverty Ratios, gaps before and after purchasing the six regimens as calculated using micro-data from the National Sample Survey, Round 68, India** | | |
| --- | --- | --- |
|  |  |  |
|  | **Rural % [95% CI]** | **Urban % [95% CI]** |
| **Baseline Poverty** |  |  |
| Ratio | 30.88 [29.93, 31.83] | 26.76 [25.61, 27.91] |
| Gap | 6.63 [6.58, 6.67] | 6.75 [6.70, 6.81] |
| **Poverty Incidence** |  |  |
| Regimen 1 | 31.22 [30.27, 32.17] | 26.90 [25.75, 28.05] |
| Regimen 2 | 34.05 [33.06, 35.03] | 28.04 [26.88, 29.20] |
| Regimen 3 | 41.05 [40.04, 42.05] | 31.14 [29.94, 32.35] |
| Regimen 4 | 55.79 [54.81, 56.77] | 38.14 [36.87, 39.41] |
| Regimen 5 | 81.18 [80.50, 81.86] | 57.75 [56.47, 59.02] |
| Regimen 6 | 80.79 [80.10, 81.47] | 57.33 [56.05, 58.61] |
| **Poverty Gap Index** |  |  |
| Regimen 1 | 6.66 [6.39, 6.93] | 6.82 [6.46, 7.18] |
| Regimen 2 | 7.70 [7.40, 7.99] | 7.42 [7.04, 7.80] |
| Regimen 3 | 10.77 [10.41, 11.13] | 9.10 [8.66, 9.54] |
| Regimen 4 | 20.14 [19.62, 20.65] | 13.72 [13.13, 14.30] |
| Regimen 5 | 61.00 [60.12, 61.88] | 33.15 [32.12, 34.18] |
| Regimen 6 | 59.85 [58.97, 60.73] | 32.59 [31.57, 33.61] |
| **Increase in Poverty** |  |  |
| Regimen 1 | 0.34 [0.25, 0.44] | 0.14 [0.08, 0.20] |
| Regimen 2 | 3.17 [2.83, 3.51] | 1.28 [1.08, 1.49] |
| Regimen 3 | 10.17 [9.62, 10.72] | 4.38 [4.00, 4.77] |
| Regimen 4 | 24.91 [24.13, 25.69] | 11.38 [10.76, 12.00] |
| Regimen 5 | 50.30 [49.40, 51.20] | 30.99 [30.00, 31.97] |
| Regimen 6 | 49.91 [49.01, 50.81] | 30.57 [29.59, 31.55] |
| **Increase in Poverty Gap Index** |  |  |
| Regimen 1 | 0.03 [0.03, 0.04] | 0.07 [0.07, 0.08] |
| Regimen 2 | 1.16 [1.13, 1.20] | 0.68 [0.65, 0.71] |
| Regimen 3 | 4.23 [4.12, 4.35] | 2.35 [2.26, 2.45] |
| Regimen 4 | 13.60 [13.31, 13.89] | 6.97 [6.72, 7.23] |
| Regimen 5 | 54.47 [53.76, 55.17] | 26.41 [25.68, 27.14] |
| Regimen 6 | 53.32 [52.62, 54.01] | 25.85 [25.12, 26.57] |

Note: Micro data obtained from the National Sample Survey, Round 68, Modified Mixed Reference Period (MMRP) data. Figures rounded to the second decimal place.
